# Cross-lingual hate speech detection using domain-specific word embeddings

**DOI:** 10.1371/journal.pone.0306521

**Published:** 2024-07-30

**Authors:** Ayme Arango Monnar, Jorge Perez Rojas, Barbara Polete Labra

**Affiliations:** 1 Computer Science Department, Universidad de Chile, Santiago de Chile, Chile; 2 Cero.ai, Santiago de Chile, Chile; 3 Amazon, Washington, DC, United States of America; Indian Institute of Technology Patna, INDIA

## Abstract

THIS ARTICLE USES WORDS OR LANGUAGE THAT IS CONSIDERED PROFANE, VULGAR, OR OFFENSIVE BY SOME READERS. Hate speech detection in online social networks is a multidimensional problem, dependent on language and cultural factors. Most supervised learning resources for this task, such as labeled datasets and Natural Language Processing (NLP) tools, have been specifically tailored for English. However, a large portion of web users around the world speak different languages, creating an important need for efficient multilingual hate speech detection approaches. In particular, such approaches should be able to leverage the limited cross-lingual resources currently existing in their learning process. The cross-lingual transfer in this task has been difficult to achieve successfully. Therefore, we propose a simple yet effective method to approach this problem. To our knowledge, ours is the first attempt to create a multilingual embedding model specific to this problem. We validate the effectiveness of our approach by performing an extensive comparative evaluation against several well-known general-purpose language models that, unlike ours, have been trained on massive amounts of data. We focus on a zero-shot cross-lingual evaluation scenario in which we classify hate speech in one language without having access to any labeled data. Despite its simplicity, our embeddings outperform more complex models for most experimental settings we tested. In addition, we provide further evidence of the effectiveness of our approach through an ad hoc qualitative exploratory analysis, which captures how hate speech is displayed in different languages. This analysis allows us to find new cross-lingual relations between words in the hate-speech domain. Overall, our findings indicate common patterns in how hate speech is expressed across languages and that our proposed model can capture such relationships significantly.

## 1 Introduction

This article uses words or language that is considered profane, vulgar or offensive by some readers. Due to the topic studied in this article, quoting offensive language is academically justified but we nor PLOS in no way endorse the use of these terms. Likewise, the terms do not represent the opinions of us or that of PLOS, and we condemn online harassment and offensive language.

Timely information dissemination and other types of human communications take place on the Web, especially on online social media platforms. Along with many useful information exchanges, there are also manifestations of communication disorders such as *fake news* and *hate speech*. which can produce harmful side effects. In particular, hate speech can be understood as language that expresses prejudice against particular groups of people. It is a phenomenon related to human behavior that spans cultures and languages and can seriously limit the participation of certain groups in social media activity.

Existing solutions for multilingual hate speech detection are considerably narrow, mostly because hate speech research has been primarily in English [1–4]. As a consequence, there is a considerable scarcity of labeled data, lexical resources, and models beyond the English scope. There are some recent efforts to systematically address multilingual aspects of hate speech detection, most of which rely on emerging multilingual tools such as general-purpose text representations [5–7]. However, as an emergent topic, there is still no consensus on how to undertake this issue effectively in low-resource languages. In this regard, our work focuses on investigating solutions that can leverage data from high-resource languages to improve performance for other languages with little or no resources. In particular, we address *zero-shot multilingual learning*. where there are no resources directly available for learning a task in a particular target language; hence a different language needs to be used for this purpose.

We hypothesize that general-purpose multilingual word embeddings do not necessarily capture patterns that naturally arise when words are used with a hateful intent. For instance, words related to nationality, religion, and race will mostly have a neutral connotation in general written text. Nevertheless, these same words can be loaded with hateful meanings when they are used in the text that contains hate speech [8]. Following this intuition, we propose a set of *multilingual word embeddings* that have been specifically created for hate speech. To achieve this, we created different hate speech word embedding feature spaces in different languages and aligned them in an unsupervised way using a projection technique [9].

We evaluated the effectiveness of hate speech detection of our embedding model about other general-purpose representations, using them as input features for classification in English, Spanish, and Italian.

Our findings show that the use of our hate-specific representations mostly improved cross-lingual classification model performance compared to the other representations.

In addition, we introduce a qualitative exploratory analysis of *word contexts* for our hate-specific embeddings.

This suggests that besides the information provided by translating the general meaning of words to different languages, there are more specific cross-cutting patterns in how hate speech is displayed in those languages.

For instance, for a general-purpose multilingual embedding, the natural (context-based) translation for the Italian word *“migranti”* is *“migrants”* in English and *“migrantes”* in Spanish. In contrast, the translation in our hate-specific embeddings is *“illegals”* in English and *“palestinos”* in Spanish.

These patterns allow us to transfer knowledge from one language to another when detecting hate speech. Moreover, our approach requires very little data in contrast to other representations which have been trained on massive amounts of text.

### Problem

Existing multilingual hate speech detection solutions predominantly rely on general-purpose embeddings, often trained in English. In contrast, low-resource languages (in the hate speech detection task) like Spanish remain under-treated. This study addresses the gap by introducing domain-specific multilingual word embeddings for hate speech. These low-dimensional embeddings are computationally cheap to use and perform competitively with general-purpose ones and transformer-based models.

### Research questions

RQ1: How effective are existing multilingual hate speech detection solutions, considering they are based on general-purpose embeddings and pre-trained models predominantly trained in English?
RQ2: What is the comparative effectiveness of low-dimensional hate-specific embeddings in cross-lingual hate speech detection, as opposed to other general-purpose representations and large pre-trained models, in English, Spanish, and Italian?
RQ3: How do hate-specific multilingual embeddings contribute to capturing non-traditional translations of words?

### Contributions

The following are our main contributions:

1. We introduce the first domain-specific multilingual word embeddings for hate speech classification. The main characteristic of these embeddings is their simplicity, making them easily replicable for other languages.
2. We present a comprehensive quantitative evaluation of different approaches for cross-lingual hate speech detection, demonstrating the effectiveness of our proposal.
3. We propose a strategy for qualitatively evaluating these hate speech multilingual embeddings by exploring words’ contexts. The relationships we found support the hypothesis that the context of words in a hateful scenario is different from that in a general scenario.

### Reproducibility

All the code, experiments, and resources will be publicly available in the form of a repository.

### Organization of the paper

We first describe the relevant related work in Section 2. In Section 3. we describe our approach to creating hate speech word embeddings. In Section 4. we present a quantitative evaluation of monolingual and cross-lingual hate speech detection. In Section 5. we describe our qualitative evaluation and results. Section 6 presents a summary of findings and conclusions.

### Warning

Because of the topic of our research, certain examples contain potentially offensive terms.

## 2 Related work

In this section, we review works related to hate speech detection as well as methods for word embedding projections.

### 2.1 Monolingual hate speech detection

As for other NLP tasks, English has been the most addressed language in hate speech detection. In the related literature, several methods are leveraged for in-domain English evaluation. Some of these approaches, mainly in the early years of task development, use traditional machine-learning models [3, 10, 11] such as *Support Vector Machine (SVM)*. *Logistic Regression (LR)*. and *Random Forest (RF)*. These algorithms are commonly applied using existing software tools such as *WEKA*(https://www.cs.waikato.ac.nz/ml/weka/), *scikit-learn* (http://scikit-learn.org/stable/index.html), and combined engineered features [12, 13].

Deep-learning methods have gained popularity in recent years for addressing the task, in addition to the conventional machine-learning algorithms. Convolutional Neural Networks (CNNs) [14, 15] and Recurrent Neural Networks (RNNs) [1, 2, 16, 17] are popular architectural choices for detecting hate speech. Models such as BERT [18]. which are based on Transformers, have shown success in various NLP tasks [19–22]. BETO (Spanish BERT) [23]. Italian BERT [24]. Chinese BERT [25]. and various others have been developed to facilitate progress in different languages.

In addition, there have been critical analyses of English-based systems and datasets to provide a better understanding of the problem and the possible biases existing in datasets and models [26–28].

### 2.2 Cross-lingual hate speech detection

As demonstrated for other tasks [29, 30]. a multilingual approach to the hate speech detection problem could help improve the state of the art for under-represented languages.

For languages with little to no labeled resources, we need an approach in which no information about the target language is used during the training process. We refer to this constrained scenario as *cross-lingual*.

Translating training and testing data into a common language is one of the strategies employed for this task [31]. Meta-information from network dynamics and text messages has been used as features in the literature [26]. This type of feature is considered language-independent as it is not directly related to the language in which the text is written. LASER [32] is a recently proposed model for producing multilingual embeddings for sentences. These embeddings have been combined with traditional machine learning models [6, 7] for cross-lingual hate speech detection. Another approach involves fine-tuning pre-trained multilingual models like BERT [33] (https://github.com/huggingface/transformers) [5, 6, 34] or XLM (https://github.com/facebookresearch/XLM) [35] on the training data.

### 2.3 Specific embeddings for the hate speech detection

There is limited research on specialized word representations and specific pre-trained models for this task, as seen in the works cited [36–38]. Similar to our work, some papers [37, 38] describe the construction of task-specific word embeddings, using techniques such as Word2Vec or GloVe to construct low-dimensional word embeddings. These works also utilize unlabeled data extracted from social networks considering specific hateful queries. Other works describe the construction of specific task word embeddings using different techniques. For example, Badjatiya et al. [1] use an LSTM model for building word embeddings from a dataset of labeled hateful tweets. There have also been efforts to adapt existing pre-trained models, as in the case of HateBERT [39]. where the authors retrained BERT using English-banned comments from Reddit.

Despite these efforts in the monolingual, mainly English scenario, current cross-lingual techniques often use general-purpose features or general pre-trained models. To our knowledge, no works are creating specialized multilingual representations (word embeddings) for this problem. Considering the particularities of the hate speech phenomenon, domain-specific representations will contribute to improving cross-lingual classification, and we focus our work on this. In contrast to established methodologies, our proposed approach distinguishes itself by addressing the challenge of multilingual hate speech classification through the construction of specific representations.

### 2.4 Projection-based multilingual word embeddings

One approach for creating multilingual embeddings is by using the so-called projection technique [40–42]. This requires resources that are relatively easy to obtain for most tasks. The idea is to linearly project two vector spaces into a common space by optimizing the relationship between dictionary-paired vectors obtained from bilingual dictionaries. The bilingual dictionaries can be induced from the data (unsupervised methods) [29, 43, 44] or provided beforehand (supervised methods) [9, 41, 45, 46].

We select this type of approach for the creation of our multilingual hate speech embeddings.

## 3 Hate-speech specific word embeddings

In this section, we describe the process of creating domain-specific word representations for hate speech in social media. Our methodology is divided into two steps:

1. the creation of domain-specific monolingual word embeddings for each separate language (detailed in Section 3.1), and
2. the alignment of the monolingual word embeddings into a single embedding space using bilingual dictionaries (detailed in Section 3.2).

We consider this to be a weakly supervised approach since it assumes the existence of i) a *bilingual dictionary*. of at least a few terms, to go from one language to another, and ii) a small *hate speech lexicon* for each target language. In particular, dictionaries allow us to forgo the need for large amounts of parallel or labeled data [47]. We use an off-the-shelf dictionary (described in Section 4.2).

Another consideration is the construction of monolingual word embeddings requires a significant amount of unlabeled data. However, in comparison to the huge volume of data that is needed to train general-purpose word representations, our required data is quite small. Moreover, unlabeled data can be retrieved easily from social media using a small set of domain-specific (i.e., hateful) seed terms. We detail each step next.

### 3.1 Domain-specific monolingual embeddings

We describe the creation of monolingual word embeddings from social media. First, using a set of *seed* terms (or queries), we retrieve a set of social media text messages. In particular, we use the online social network Twitter, which is a microblog (i.e., short-text-based) platform. Our seeds are based on terms contained in public lexicons of hateful words [11, 48]. These seeds guarantee that the retrieved messages contain hateful terms, causing, as an overall effect, a higher probability for hateful messages to appear in the resulting corpora. We focus on English, Spanish, and Italian. Specifically, we collected 30 million tweets in English and 10 million tweets in Spanish and Italian, each.

Using this corpus, we train Word2Vec [49] 100-dimensional word embeddings for each language individually. By applying the Word2Vec technique, we obtain (mathematical) close vectors for semantically similar words. Since our corpus is biased towards hateful content—thanks to the weak supervision provided by the seed terms—we consider our resulting embeddings to be domain (hate) specific. Notice that the previous result proceeds for any other algorithm for creating the monolingual embeddings.

### 3.2 Alignment of monolingual spaces

In this section, we describe how different monolingual word embeddings are aligned into a single embedding space. As an alignment algorithm, we adopt a technique based on canonical correlation analysis (CCA) proposed by Faruqui and Dyer [9]. In this process, a pair of monolingual word vectors are projected into a common space by learning two projection matrices *V* and *W* that maximize the correlation between the dictionary-paired projected vectors.

More specifically, assume that $X\in R^{d\times n_{1}}$ and $Y\in R^{d\times n_{2}}$ are two word-embedding matrices corresponding to two different languages, where every embedding is a column in each matrix. We note that *n*_1_ and *n*_2_ might be different as the sets of embeddings might be created from vocabularies of different sizes.

#### 3.2.1 Canonical variables for embeddings

Further, assume that we have a set of *n* translated terms between the two languages, and let *X*′ and *Y*′ be matrices in $R^{d\times n}$ that is obtained by taking the columns from *X* and *Y*, respectively, that correspond to the aligned translated terms.

The canonical variables for set *X*′ are denoted as *U* = [*U*_1_, *U*_2_, …, *U*_*p*_], and for set *Y*′, *V* = [*V*_1_, *V*_2_, …, *V*_*q*_]. The canonical variables are linear combinations of the original embeddings:

$$\begin{array}{c} U_{i}=\sum_{j=1}^{p}a_{ij}X_{j}^{\prime } \end{array}$$

$$\begin{array}{c} V_{j}=\sum_{k=1}^{q}b_{kj}Y_{k}^{\prime } \end{array}$$

Here, *a*_*ij*_ and *b*_*kj*_ are the coefficients to be determined through the canonical correlation analysis.

#### 3.2.2 Objective function

The goal is to maximize the correlation between the canonical variables *U* and *V*.

$$\begin{array}{c} \left(U,V\right)\;=\;\underset{U,V}{\text{arg}\;\text{max}\;}\rho \left(UX^{\prime },VY^{\prime }\right) \end{array}$$

#### 3.2.3 Projecting embeddings

Once the canonical correlation analysis is performed, the canonical variables *U* and *V* provide the projections of the original embeddings into a shared subspace.

Using these two projection matrices, we can project the entire set of embeddings for both languages to obtain our final set of embeddings

$$\begin{array}{c} X^{m}=UX,\;\;Y^{m}=VY \end{array}$$

With this method, we have aligned our initial word embeddings from step 1 (Section 3.1) into a single vector space. Following the described process, it is possible to replicate the algorithm for different languages given a set of hateful seeds. The hateful lexicon Hurtlex contained terms in 50 languages with basic computational resources. We include an implementation of the complete pipeline in our code repository.

## 4 Experiments and results

The goal of our experiments is to evaluate quantitatively and qualitatively the performance of our hate embeddings in comparison to existing general-purpose word representations. Specifically, we evaluate different settings using multilingual embeddings for English, Spanish, and Italian.

### 4.1 Datasets for evaluation

We used three labeled Twitter hate speech datasets for our experiments. A summary of these datasets is presented in Table 1. Each dataset is detailed next:

*English dataset:* This dataset consists of the English dataset by Arango et al. [26]. created in 2019, and the one created for SemEval in 2019 by Basile et al. [50]. Both datasets contain hate speech against immigrants and women. They originated in the United States; therefore, hate targets, as well as specific terms, are framed within that particular cultural context.
*Italian dataset:* This dataset is composed of the dataset by Sanguinetti et al. [52]. which is part of the “Hate Speech Italian Monitoring Program”. The hate targets are women and immigrants.
*Spanish dataset:* This dataset consists of the dataset by Pereira et al. [51]. which contains hate speech related to racism, sexism, and xenophobia. Additionally, we used the Spanish portion of the SemEval 2019 dataset by Basile et al. [50]. which includes hate speech against immigrants and women. The tweets originated in Spain.

**Table 1:** Description of the datasets used in our evaluation. For each dataset, we show the number of tweets per class.

Lang	Dataset	Hate	Non-Hate	Total
EN	Arango et al. [26]	2,920	4,086	7,006
	Basile et al. [50]	5,390	7,415	12,805
ES	Basile et al. [50]	2,228	3,137	5,365
	Pereira et al. [51]	1,567	4,433	6,000
IT	Sanguinetti et al. [52]	1,291	5,637	6,928
Total		13,396	24,708	38,104

Datasets within the same language were merged, and their labels were binarized, following a commonly used strategy in this area for creating larger collections [6, 53].

### 4.2 Bilingual dictionary

We use a bilingual dictionary consisting of word-aligned pairs from the Hurtlex lexicon [48]. Hurtlex is a multilingual lexicon that we use to match hateful terms between different languages. It has been successfully used for cross-domain hate speech detection [54, 55]. The dictionaries comprise more than ten thousand words typically used in the hate speech domain and their translations to a second language. The particularity of Hurtlex is that it includes terms with different colloquial equivalents that are not usually included in generic dictionaries, as well as words that could typically appear in hateful content. An example comparing the diversity of Hurtlex with the Muse dictionary can be found in our code repository. According to Shakurova et al. [56]. better results are obtained when the bilingual lexicon is from the specific domain of the task.

### 4.3 Quantitative evaluation

Overall, we study cross-language classification in a transfer learning scenario, when the classifier is trained on labeled data for one language and then is used to classify in another.

The Algorithm 1 describes the experimental process. We used the three datasets described in Section 4.1 in languages English (*EN*), Spanish (*SP*), and Italian (*IT*). For each of the possible training testing combinations of the three datasets (*SETUPS*), we compare different multilingual embeddings (*EMBEDDINGS*) including our proposal. For performing the comparison we tested several models (*MODELS*) and reported the best performance by setup and by word embedding (*RESULTS*). In Tables 2 and 3 we show monolingual and cross-lingual results. Next, we detail every section of the process.

**Table 2:** Experimental results for monolingual hate speech detection. The cells show the F1 score of the best model for each combination of setup and input representation (embedding). The bold numbers represent the best score per setup.

Setup	Fine-tuned-BERT	Input Representations				
		mBERT	MUSE	HateEmb	LASER	XLM
*EN* → *EN*	73.45	**74.51**	70.26	73.59	67.17	74.35
*ES* → *ES*	73.45	70.74	67.57	69.82	63.81	**75.83**
*IT* → *IT*	69.30	69.74	63.95	69.38	77.48	**73.25**

**Table 3:** Experimental results for cross-lingual hate speech detection. The cells show the F1 score of the best model for each combination of setup and input representation (embedding). The bold numbers represent the best score per setup.

Setup	Input Representations				
	MUSE	Laser	mBert	XLM	HateEmb
*EN* → *SP*	50.73	52.25	**61.45**	60.48	57.48
*IT* → *SP*	46.22	54.70	60.19	**65.43**	56.28
*EN* → *IT*	55.40	54.87	58.36	49.13	**60.33**
*SP* → *IT*	53.81	55.09	54.37	56.40	**59.74**
*SP* → *EN*	48.19	53.76	**57.72**	50.03	56.58
*IT* → *EN*	43.29	53.68	55.27	50.63	**56.33**

***SETUPS.*** We test the nine possible source and target language combinations (e.g. *SP* → *IT*).

Each dataset described in Section 4.1 is split into three sets: training (80%), testing (10%), and validation (10%) for adjusting the hyper-parameters of the models. Even though our main interest is the cross-lingual scenario, we also include monolingual experiments to have reference performance of models. Intuitively, the closer the cross-lingual results are to the monolingual ones, the better they are at transferring knowledge from one language to another.

Multilingual embeddings can be used in monolingual scenarios, although their aligned characteristic are not useful in this case.

**Algorithm 1** Experimental process for comparing our hateful embeddings with general purpose word embeddings.

1: **SETUPS** = [*ES* → *ES*, *EN* → *EN*, *IT* → *IT*, *EN* → *ES*, *EN* → *IT*, *ES* → *EN*, *ES* → *IT*, *IT* → *EN*, *IT* → *ES*];

2: **EMBEDDINGS** = [LASER, MUSE, mBERT, **HATE_EMB**];

3: **MODELS** = [LR, XGB, SVM, RF, DT, NB, CNN, FNN, LSTM, MHATTN, LSTMCNN, LSTMATTN];

4: **ALL_RESULTS** = {};

5: **for** setup in **SETUPS do**

**6:**  **train, val, test** = *get*_*partitions*(**setup**)

**7:**  **for emb in EMBEDDINGS do**

**8:**   **best_result = 0**

**9:**   **for model in MODELS do**

**10:**   **best_model** = *h*_*tunning* **(emb, model, train, val)**

**11:**    **result** = *test* **(emb, best_model, train, test)**

**12:**    **if result** > **best_result then**

**13:**     **best_result = result**

**14:**    **end if**

**15:**   **end for**

**16:**   **ALL_RESULTS[setup][emb] = best_result**

**17:**  **end for**

**18:**  **if** *is*_*monolingual* **(setup) then**

**19:**   **BERT** = *get*_*BERT* **(setup)**

**20:**   **best_BERT** = *fine*_*tunning* **(BERT, train, val)**

**21:**   **result** = *test* **(best_BERT, train, test)**

**22:**   **ALL_RESULTS[setup][fine_tuned_BERT] = result**

**23:**  **end if**

**24:** **end for**

**25:** **Return ALL_RESULTS**

***EMBEDDINGS***. We consider five types of multilingual embeddings: MUSE [29]. a set of general embeddings aligned for multilingual contexts; BERT [18] and XLM [57]. general purpose pre-trained models for NLP that can be used to produce embeddings for sentences (sequences of words); LASER [32]. a recent model for producing multilingual sentence embeddings and HATE_EMB our proposed embeddings described in Section 3.

In the cross-lingual setups, we use multilingual BERT (mBERT for short). In addition, we use BERT pre-trained with monolingual data Italian [24]. Spanish [58] and English [18]. those for performing fine-tuning in the specific task of hate-speech detection. We evaluate the usefulness of these three representations by using them to generate input features for several classification models. At the same time, they perform as baselines for comparing the hate embeddings.

***MODELS***. We evaluate several traditional machine learning models including Logistic Regression (LR), XGBoost (XGB), Support Vector Machines (SVM), Random Forest (RF), Decision Trees (DT), and Naive Bayes (NB) classifiers. We also incorporate deep learning models, including Convolutional Neural Networks (CNN), Feedforward Neural Networks (FNN), Long Short-Term Memory Networks (LSTM), and Multi Head Attention (MHATTN). In addition, we combine LSTM and CNN layers (LSTMCNN), as well as LSTM with Attention [59] (LSTMATTN). We tuned these models to find the best possible values for the different hyperparameter combinations (e.g. batch size, learning rate, dimensions, number of layers, etc.). The complete grid search and the best resulting parameters will be described in a section of our code repository. In the case of monolingual setups, we perform fine-tuning for the pre-trained BERT models according to the setup. case, the BERT model serves as input representation and classification model (BERT Italian [24]. BERT Spanish [58] and BERT English [18]).

### 4.4 Results

Next, we detail our results for the monolingual and cross-lingual classification task, as described in Section 4.3.

#### Monolingual results

Our embeddings (HateEmb) show competitiveness compared to more complex input representations. They outperform MUSE embeddings in all configurations. Additionally, the results differ from those using mBERT by less than 1%.

In Table 2. we present the results (in terms of F-score) obtained in monolingual evaluations for each setup and embedding representation. The F1 score corresponds to the best result obtained for each experimental setup and embedding representation independently of the model used.

Moreover, we include the results of fine-tuning the corresponding BERT model (Fine-tuned BERT).

In monolingual experiments, for the three datasets we considered, the transformer-based models XLM and BERT show the best performances.

Another important observation is that hate embeddings yield similar results compared to the BERT multilingual embeddings. Considering the state-of-the-art language models, embedding models were trained with a massive amount of data and the learning process included a higher number of parameters. We believe that our hate embeddings show encouraging results.

In general, the Italian experiments yield the worst results, as expected, since we have less data available in this language.

#### Cross-lingual results

The hate embeddings (HateEmb) show the best results, outperforming the general-purpose ones in three of the six experimental setups. This is noteworthy since hate embeddings are created with very little data in comparison to the other more complex models. Table 3 shows the results of the cross-lingual experiments using several different input representations.

As we have mentioned, this setting is called zero-shot multilingual transfer learning, since no data from the target language is used during training. This is, arguably, the most challenging multilingual transfer learning task. Our proposed hate embeddings outperformed LASER and Muse embeddings in all configurations. Muse was constructed with a similar approach, aligning monolingual spaces. The improvement in the performance of HateEmb can be explained by the nature of the data used in training the monolingual vectors and the specialized bilingual dictionary.

Additionally, the hateful embeddings achieved the best performance in three of the six configurations and were outperformed by the BERT model and XLM when used to generate the input representations.

In those cases, our approach ranked second best. We consider this to be a good result, given that BERT and XLM are huge models that require training millions of parameters and specialized hardware. In contrast, our embeddings are extremely lightweight and can be trained on general-purpose machines.

One of the possible reasons for our embeddings to outperform more sophisticated ones is that they are trained specifically on social media text containing hate speech. In contrast, general-purpose embeddings like MUSE are trained on diverse corpora that may not capture the nuances of hate speech well. This domain-specific training allows hate embeddings to capture subtle linguistic cues specific to hate speech, leading to better performance in hate speech detection tasks. General-purpose embeddings trained on diverse corpora may contain noise from non-hate speech contexts, which can degrade performance in hate speech detection tasks. Hate embeddings, trained on hate speech data, are less likely to suffer from this noise, resulting in improved accuracy.

In addition, the hate embeddings are aligned using a bilingual dictionary specifically tailored for hate speech, such as Hurtlex. This allows hate embeddings to capture cross-lingual semantic information relevant to hate speech, leading to improved performance in cross-lingual hate speech detection tasks compared to general-purpose embeddings.

## 5 Qualitative evaluation

The intrinsic quality of multilingual word embeddings is usually evaluated based on the Bilingual Lexicon Induction (BLI) task [60]. This task measures how close the vectors representing translations in different languages are to each other. BLI relies on nearest neighbor search, identifying the most similar word in the target language given a word in a source language. The target and source words are expected to be translations of each other according to a validation dictionary. Over these results can be calculated quantitative metrics like precision and recall [29].

There are several difficulties in directly applying a BLI-like quantitative approach to assess the intrinsic quality of our embeddings. The main difficulty is that hate speech is a problem where word meanings often extend well beyond their literal translations. Thus, a low BLI score for general terms does not necessarily mean low quality for hate speech detection. We present a custom qualitative analysis based on the idea of BLI-like tests.

As a result of our qualitative evaluation, new cross-lingual relations between words emerged.

### 5.1 Cross-lingual relations in vectors spaces

BLI inspires our first qualitative evaluation, extracting the most related terms across different languages given a seed term. This exercise aims to identify equivalent hateful meanings rather than direct translations in both vector spaces and within the labeled datasets.

In Table 4. we present a sample of the relationships between terms comparing the hate embeddings (HateEmb) with the general-purpose multilingual embedding MUSE.

**Table 4:** Terms extracted from the datasets and the 1-nearest neighbors in vector spaces of different languages.

Seed	HateEmb NN		Muse NN	
IT	EN	ES	EN	ES
migranti	illegals	palestinos	migrants	migrantes
terroristi	cockroaches	protestantes	terrorists	terroristas
islamici	islamic	sionista	islamic	islamicos
ES	EN	IT	EN	IT
gitano	portuguese	negro	gypsy	gitano
fachas	bigots	comunisti	—	—
negros	blacks	neri	blacks	neri
EN	ES	IT	ES	IT
retarded	imbecil	idiota	mental	ritardato
muslims	musulmanes	musulmani	musulmanes	musulmani
christians	catolicos	cattolici	cristianos	cristiani

We manually selected these terms to represent groups that have been targets of hate, ensuring they were not in the bilingual dictionary used for embedding alignment. This allowed us to display a new relationship.

For each selected *source term*. we show the nearest neighbors (NN) terms in a language different from the source language. In most cases, the nearest neighbors in the MUSE space are terms whose standard meanings are the same in both languages. For example, in Table 4. for the Italian word “migranti”, we found that the nearest terms in the MUSE space are “migrants” in English and “migrantes” in Spanish. These are literal translations for the three languages. On the other hand, the nearest neighbors in the hate-specific embedding space are “illegals” (English) and “palestinos” (Spanish).

We can observe that these are neither direct nor neutral translations of the original word “migranti”. However, these words are likely to appear in similar contexts for hateful text. Within this scenario, the term “illegals” is used commonly to refer to a person who migrates to the U.S. illegally. Similarly, the word “palestinos” (that means a person of Palestine origin) is associated in hate-related contexts to a person who is an immigrant from an Arabian country.

We argue that evaluating the hate embeddings considering literal translations is not suitable since in the hateful content, words like “migranti” could acquire different meanings. The correct equivalence that should have been found is unknown, due to the complexity of the hate speech problem. Moreover, expecting the same relationships across different languages (e.g. “migrants”—“terrorist” = “migrantes”—“terroristas”) is not correct either. The targets of hate in different languages are different depending on the socio-cultural scenario. In most of the cases, we were able to observe non-trivial translations when exploring our hate embeddings. However, in a few cases, we could observe that the equivalences are the same as in MUSE (e.g. *“negros”* in Spanish, as *“blacks”* in English and *“neri”* in Italian). More experimentation is needed to derive a more robust conclusion, but our qualitative results provide positive evidence that our domain-specific embeddings capture non-trivial meanings and translations.

### 5.2 Cross-lingual relations in labeled datasets

In this section, we introduce a method for qualitatively exploring the ability of our embeddings to capture equivalences between hateful concepts in different languages over a labeled dataset. In the previous section, we use similarity measures (nearest neighbors) over the general embedding space, considering all the vocabulary used to construct those embeddings (unlabeled general data). In this section, we focus exclusively on texts from the positive class of hate speech labeled datasets in different languages. That is, we focus on multilingual data that we know that contains hateful information. We use the hate embeddings plus *association rules* to devise a similarity measure among terms in different languages. This experiment would serve as an intrinsic qualitative evaluation as we can assess how good the translations obtained for simple hateful terms. We next explain in more detail the method we devised to obtain the equivalences.

#### 5.2.1 Association rules and word contexts

For the first step, let *x* be a word and *U* a set of words all from one of the labeled datasets. From each dataset, we extract association rules of the form {*x*}⇒*U* with the following semantics: if *x* occurs in a text *T*, then *U* ⊆ *T* with certain confidence [61]. In that way, we can find words that usually occur together in the same text. We extract rules for the top most frequent terms *x* in each dataset and impose lower bounds in *confidence* and *support*.

The *support* measures how often a specific item-set or item appears in a dataset, or its level of popularity. A higher value for support means that the itemset or item occurs more frequently within the dataset. *Confidence* refers to the degree of reliability or strength in the relationship between two itemsets or items. This measure is determined by comparing the number of transactions (tweets in our scenario) that contain both the antecedent and consequent itemsets to the number of transactions that contain only the antecedent. A higher confidence value suggests a more significant correlation between the two itemsets or items. [62].

We note that one can obtain many different association rules in each dataset and for each frequent term *x*. Using all the rules with the form {*x*}⇒*U*_*i*_, we compute *the context* of the word *x* as *C*(*x*) = ⋃*U*_*i*_. Our similarity measure for two words is based on the similarity of contexts for those words as we next explain.

#### 5.2.2 Context-based similarity measure

We still need to introduce some additional notation to present our similarity measure. For every word *u* ∈ *C*(*x*) we denote by *supp*_*x*_(*u*) and *conf*_*x*_(*u*) the *support* and *confidence* of term *u* in the association rule of the form {*x*}⇒*U* it appears. Given words *u* and *v*, appearing in contexts say *C*(*x*) and *C*(*y*), respectively, we define the following expression that essentially compares their support and confidence metrics in their respective contexts:

$$\begin{array}{c} 1-|{supp}_{x}\left(u\right)-{supp}_{y}\left(v\right)|/2+|{conf}_{x}\left(u\right)-{conf}_{y}\left(v\right)|/2 \end{array}$$

We call this expression the *metrics similarity* between *u* and *v* and we denote it by *met- sim*(*u*, *v*). We combine the above similarity for context words with a usual embedding similarity based on cosine similarity by averaging both to obtain a combined similarity:

$$\begin{array}{c} sim\left(u,v\right)=(cos-sim(u,v)+met-sim(u,v))/2 \end{array}$$

That is, we give the same importance to how similar the vectors are (*cos-sim*), and how similar the importance (confidence and support) of the association rules they appear in are (*met-sim*).

We now have all the ingredients to define the *context similarity* of words. Let *x* and *y* be words with contexts *A* = *C*(*x*) and *B* = *C*(*y*), respectively. Then, their context similarity, denoted by *cont- sim*(*x*, *y*) is defined as

$$\begin{array}{c} cont-sim\left(x,y\right)=\frac{1}{2}(\underset{u\in A}{\text{mean}}(\underset{v\in B}{\text{max}}sim\left(u,v\right))+\underset{v\in B}{\text{mean}}(\underset{u\in A}{\text{max}}sim\left(u,v\right))) \end{array}$$

That is, for every word in *x*’s context (*A*), we compute its maximum similarity with words in *y*’s context (*B*) and take the mean over all those similarities, and the other way around. The results of both directions are averaged.

#### 5.2.3 Context-based similarity for hateful words

For each dataset of every language, we first selected some frequent words (*seed terms*) appearing in the hateful-labeled texts. Then, for each seed, we selected the words that are more (context-) similar to all the words appearing in hateful labeled texts in a different language. Table 5 shows examples of terms and the top two most similar words using our hate-specific embeddings and Table 6 shows the results for a similar experiment but using MUSE embeddings. As a comparison, the tables also show a similar experiment for the non-hate texts. More examples can be found in our repository.

**Table 5:** For some terms, we found the top most similar ones in different languages using the HateEmb embeddings for hateful and non-hateful classes. We can observe different relations depending on the nature of the expressions. The numbers represent the similarity achieved in each case (%).

Seed	Hateful Data		No-Hateful Data	
EN	ES	IT	ES	IT
girls	perra-73	immigrati-73	cosas-69	dire-68
	igual-73	fatto-71	subnormal-68	arriva-68
muslims	putos-72	islamica-74	migrantes-70	cristiani-69
	fascistas-71	italia-72	guerra-69	europa-69
ES	EN	IT	EN	IT
gitano	invaders-66	vivere-74	fingers-61	caccia-74
	mexican-65	male-73	loving-61	guida-74
palestinos	disaster-64	cancro-79	iraqi-64	accordo-73
	genocide-62	pericolo-78	fleeing-64	mette-73
IT	EN	ES	EN	ES
migranti	refugees-72	sudacas-82	actually-72	cosas-82
	living-74	podemos-82	something-71	normal-81
terroristi	muslims-70	fascistas-80	actually-72	ridículo-82
	welcome-69	putos-79	without-72	cosas-82

**Table 6:** Results for an experiment similar to the one presented in Table 5. But considering the general-purpose MUSE multilingual embeddings instead of our hate-specific embeddings. We can observe different relations depending on the nature of the expressions. The numbers represent the similarity achieved in each case (%).

Seed	Hateful Data		No-Hateful Data	
EN	ES	IT	ES	IT
girls	gusta-75	islamico-71	mamón-70	vedere-69
	mujeres-74	stranieri-71	eres-70	bene-68
muslims	españa-76	ecco-76	guerra-73	pero-76
	musulmanes-72	italia-75	llegan-71	europa-76
ES	EN	IT	EN	IT
gitano	hopefully-70	credo-68	cock-68	cambiare-66
	complete-70	pensa-68	gotta-67	stranieri-66
palestinos	beaners-61	bestie-67	return-69	largo-69
	disaster-61	bestie-67	syrian-68	corridoi-69
IT	EN	ES	EN	ES
migranti	refugees-73	refugiados-72	migrants-74	mamón-74
	living-72	quiere-70	take-71	catalán-71
terroristi	religion-75	malditos-73	migrants-73	viendo-72
	quran-75	mientras-724	ready-72	mamón-72

Even though the labeled datasets are relatively small and from specific types of hate, we still find interesting cross-lingual relations. As expected, these relations are different depending on the nature of the text (hateful versus non-hateful).

For example, using hate-embeddings, for the Italian word “terroristi”, which is a neutral translation of the English “terrorists”, we found the words “muslims” and “fascistas” (see Table 5). In particular, “fascistas” is an adjective related to fascism and is used pejoratively. Another example that illustrates the results of using hate-embeddings is that the most similar word to “girls” in English was “perra” for Spanish. This last term is a very demeaning way to refer to women in that language.

On the other hand, for Muse embeddings (see Table 6) the closest term to “girl” is “gusta” (like) which is not semantically related. In addition, the most similar term to “gitano”, which means gypsy in Spanish, was “invaders”. However, using Muse embeddings the closest word is “hopefully”, which is not meaningfully related.

The relationships that we have found using our hate embeddings can be interpreted as a cross-cultural similarity in how concepts are related to each other within hateful contexts. Furthermore, these relationships, although only qualitative, are very difficult to find when we repeat this experiment on general-purpose multilingual embeddings.

### 5.3 Limitations

CCA, the method used for aligning the word embeddings into a multilingual space, relies on two resources: unlabeled data for constructing monolingual embeddings and bilingual dictionaries for the alignment process. The first resource is relatively easy to obtain, but bilingual dictionaries may be unavailable for certain languages.

The dictionary used in this paper, Hurtlex, contains equivalences relative to the hate speech phenomenon in 50 languages. However, the technique’s effectiveness may still depend on the specific characteristics of these dictionaries, such as the number of equivalences and quality of them. However, CCA’s applicability to low-resource languages can be improved using some additional strategies. For example, refining the model after the creation of multilingual embeddings. The initial dictionary can be augmented by inferring additional bilingual equivalences from these vectors. This expanded dictionary enables another iteration of the method, allowing the process to be repeated multiple times to achieve improved embeddings.

Another limitation of projecting multilingual embeddings, as well as using pre-trained language models, is the risk of introducing biases. The training data for creating the monolingual embeddings may contain stereotypes or discriminatory content that can reinforce cultural prejudices and impact the performance of hate speech detectors. In addition, the projection techniques, relying on statistical correlations between language embeddings, may introduce a skewed representation of languages based on the bilingual dictionaries used.

## 6 Summary & conclusions

We have presented a detailed analysis of cross-lingual hate speech classification aimed at transferring knowledge from one (or more) language to another.

Although simple, our proposed technique outperformed more complex ones. Leveraging specific-domain cross-lingual resources could be a promising direction for this task, which has been largely unexplored.

We summarize our main findings as follows:

- Hate embeddings demonstrate competitive performance for monolingual classification compared to general-purpose data representations. As shown in Table 2. our embeddings outperform MUSE for all languages. Additionally, we achieve similar performance to BERT, despite requiring significantly fewer training resources than BERT.
- Hate embeddings are effective for cross-lingual classification, as shown in Table 3. They outperform other approaches in 4 out of 6 configurations: *EN* → *ES*, *EN* → *IT*, *ES* → *EN*, *IT* → *EN*. In the remaining experiments, they are the second best performing.
- Our hate embeddings enable the extraction of significant multilingual semantic relationships in hateful contexts, not limited to literal translations as with other general-purpose multilingual embeddings (Tables 4–6). This indicates that the context of words in a hateful scenario differs significantly from their context in a general scenario. Moreover, these relationships enhance currently available lexical resources.

The performance of hate embeddings compared to much more sophisticated general-purpose representations suggests that they can effectively capture domain-specific information critical for hate speech detection.

Overall, there appear to be cross-cutting patterns in hate speech that transcend languages. Furthermore, knowledge transfer from one language to another is expected to contribute to the improvement of hate speech detection models in any language, reducing the need for massive amounts of labeled data.

As future directions, we will explore other algorithms for creating domain-specific representations for hate speech. Additionally, we will study how cultural differences affect hate speech detection, even within the same language.

## References

[1] Badjatiya, Pinkesh and Gupta, Shashank and Gupta, Manish and Varma, Vasudeva. Deep learning for hate speech detection in tweets. Proc. 26th WWW Companion. 759–760. 2017

[2] Sweta Agrawal and Amit Awekar. Deep Learning for Detecting Cyberbullying Across Multiple Social Media Platforms. Proc. 40th ECIR. 141–153. 2018.

[3] Despoina Chatzakou, Nicolas Kourtellis, Jeremy Blackburn, Emiliano De Cristofaro, Gianluca Stringhini, Athena Vakali. Mean Birds: Detecting Aggression and Bullying on Twitter. Proc. ACM on Web Science Conference, WebSci. 13–22. 2017.

[4] Hosseinmardi, Homa and Mattson, Sabrina Arredondo and Rafiq, Rahat Ibn and Han, Richard and Lv, Qin and Mishra, Shivakant. Analyzing labeled cyberbullying incidents on the Instagram social network. International Conference on Social Informatics. 49–66. 2015.

[5] Lukas Stappen, Fabian Brunn, Björn W. Schuller. Cross-lingual Zero- and Few-shot Hate Speech Detection Utilising Frozen Transformer Language Models and AXEL. CoRR. abs/2004.13850.

[6] Sai Saketh Aluru, Binny Mathew, Punyajoy Saha and Animesh Mukherjee. Deep Learning Models for Multilingual Hate Speech Detection. CoRR. abs/2004.06465. 2020.

[7] Vitiugin, Fedor and Senarath, Yasas and Purohit, Hemant. Efficient Detection of Multilingual Hate Speech by Using Interactive Attention Network with Minimal Human Feedback. 13th ACM Web Science Conference 2021.

[8] Mai ElSherief, Vivek Kulkarni, Dana Nguyen, William Yang Wang, Elizabeth M. Belding. Hate Lingo: A Target-Based Linguistic Analysis of Hate Speech in Social Media. Proc. 12th ICWSM. 42–51. 2018.

[9] Manaal Faruqui and Chris Dyer. Improving Vector Space Word Representations Using Multilingual Correlation. Proc. 14th EACL. 462–471. 2014.

[10] Zeerak Waseem, Dirk Hovy. Hateful Symbols or Hateful People? Predictive Features for Hate Speech Detection on Twitter. Proc. SRW@HLT-NAACL. 88–93.

[11] Thomas Davidson, Dana Warmsley, Michael W. Macy and Ingmar Weber. Automated Hate Speech Detection and the Problem of Offensive Language. Proc. 11th International Conference on Web and Social Media 512–515. 2017.

[12] Papegnies, Etienne and Labatut, Vincent and Dufour, Richard and Linares, Georges. Graph-based Features for Automatic Online Abuse Detection. SLSP. Springer 70–81. 2017.

[13] Tahmasbi, Nargess and Rastegari, Elham. A Socio-contextual Approach in Automated Detection of Cyberbullying. Proc. 51st HICSS. 2018

[14] Gambäck, Björn, Sikdar, Utpal Kumar. Using Convolutional Neural Networks to Classify Hate-Speech. Proceedings of the First Workshop on Abusive Language Online. 85–90. 2017.

[15] Park, Ji Ho, Fung, Pascale. One-step and Two-step Classification for Abusive Language Detection on Twitter. Proc. Workshop on Abusive Language Online. 41–45 2017

[16] Ziqi Zhang, David Robinson, Jonathan A. Tepper. Detecting Hate Speech on Twitter Using a Convolution-GRU Based Deep Neural Network. 15th ESWC. 745–760. 2018.

[17] Rui Cao, Roy Ka-Wei Lee, Tuan-Anh Hoang. DeepHate: Hate Speech Detection via Multi-Faceted Text Representations. WebSci’20: 12th ACM. 11–20. 2020.

[18] Jacob Devlin, Ming-Wei Chang, Kenton Lee, Kristina Toutanova. BERT: Pre-training of Deep Bidirectional Transformers for Language Understanding. Proc. NAACL-HLT. 4171–4186.

[19] Mozafari Marzieh and Farahbakhsh Reza and Crespi Noël. Hate speech detection and racial bias mitigation in social media based on BERT model. PloS One Volume 15 Number 8. 2020. doi: 10.1371/journal.pone.0237861 32853205 PMC7451563

[20] Liu, Junhua and Singhal, Trisha and Blessing, Lucienne TM and Wood, Kristin L and Lim, Kwan Hui. Crisisbert: a robust transformer for crisis classification and contextual crisis embedding. Proceedings of the 32nd ACM conference on hypertext and social media. 133–141. 2021.

[21] Hoang, Mickel and Bihorac, Oskar Alija and Rouces, Jacobo. Aspect-based sentiment analysis using bert. Proceedings of the 22nd nordic conference on computational linguistics. 187–196. 2019.

[22] Labusch, Kai and Kulturbesitz, Preußischer and Neudecker, Clemens and Zellhöfer, David. BERT for named entity recognition in contemporary and historical German. Proceedings of the 15th conference on natural language processing, Erlangen, Germany. 8–11. 2019.

[23] Canete José. and Gabriel Chaperon and Rodrigo Fuentes and Jorge Pérez. Spanish pre-trained BERT model and evaluation data. PML4DC at ICLR. 2020.

[24] Marco Polignano, Pierpaolo Basile, Marco de Gemmis, Giovanni Semeraro, Valerio Basile. AlBERTo: Italian BERT Language Understanding Model for NLP Challenging Tasks Based on Tweets. Proc. 6th ICCL. 2019.

[25] Cui, Yiming and Che, Wanxiang and Liu, Ting and Qin, Bing and Yang, Ziqing. Pre-training with whole word masking for chinese bert. IEEE/ACM Transactions on Audio, Speech, and Language Processing. 3504–3514. 2021.

[26] Arango Aymé and Pérez Jorge and Poblete Barbara. Hate speech detection is not as easy as you may think: A closer look at model validation (extended version). Information Systems. 2020.

[27] Thomas Davidson, Debasmita Bhattacharya, Ingmar Weber. Racial Bias in Hate Speech and Abusive Language Detection Datasets. CoRR. abs/1905.12516. 2019.

[28] Maarten Sap, Dallas Card, Saadia Gabriel, Yejin Choi, Noah A. Smith. The Risk of Racial Bias in Hate Speech Detection. Proceedings of the 57th Conference of the Association for Computational Linguistics, ACL 2019. 1668–1678. 2019.

[29] Alexis Conneau, Guillaume Lample, Marc’Aurelio Ranzato, Ludovic Denoyer, Hervé Jégou. Word Translation Without Parallel Data. CoRR. abs/1710.04087. 2017.

[30] Alexis Conneau, Ruty Rinott, Guillaume Lample, Adina Williams, Samuel R. Bowman, Holger Schwenk, et al. XNLI: Evaluating Cross-lingual Sentence Representations. Proceedings of the 2018 Conference on Empirical Methods in Natural Language Processing. 2475–2485. 2018.

[31] Endang Wahyu Pamungkas, Viviana Patti. Cross-domain and Cross-lingual Abusive Language Detection: A Hybrid Approach with Deep Learning and a Multilingual Lexicon. Proc. 57th ACL. 363–370. 2019.

[32] Holger Schwenk Matthijs Douze. Learning Joint Multilingual Sentence Representations with Neural Machine Translation. Proceedings of the 2nd Workshop on Representation Learning for NLP, Rep4NLP@ACL. 2017.

[33] Pires, Telmo and Schlinger, Eva and Garrette, Dan. How multilingual is multilingual BERT?. arXiv preprint arXiv:1906.01502. 2019

[34] Juan Manuel Pérez, Aymé Arango, Franco M. Luque. ANDES at SemEval-2020 Task 12: A jointly-trained BERT multilingual model for offensive language detection. CoRR. abs/2008.06408. 2020.

[35] Ranasinghe, Tharindu and Zampieri, Marcos. WLV-RIT at HASOC 2020: Offensive Language Identification in Code-switched Texts. bookProceedings of FIRE 2020.

[36] Satyajit Kamble, Aditya Joshi. Hate Speech Detection from Code-mixed Hindi-English Tweets Using Deep Learning Models. CoRR. abs/1811.05145. 2018.

[37] Alatawi, Hind S and Alhothali, Areej M and Moria, Kawthar M. Detecting white supremacist hate speech using domain specific word embedding with deep learning and BERT. IEEE Access. 9. 106363–106374. 2021.

[38] Saleh Hind and Alhothali Areej and Moria Kawthar. Detection of hate speech using BERT and hate speech word embedding with deep model. Applied Artificial Intelligence. 2023. doi: 10.1080/08839514.2023.2166719

[39] Tommaso Caselli, Valerio Basile, Jelena Mitrovic, Michael Granitzer. HateBERT: Retraining BERT for Abusive Language Detection in English. CoRR. abs/2010.12472. 2020.

[40] Sebastian Ruder, Ivan Vulic, Anders Søgaard. A Survey of Cross-lingual Word Embedding Models. J. Artif. Intell. Res. 569–631. 2019.

[41] Tomas Mikolov, Quoc V. Le, Ilya Sutskever. Exploiting Similarities among Languages for Machine Translation. CoRR. abs/1309.4168. 2013.

[42] Goran Glavas, Robert Litschko, Sebastian Ruder, Ivan Vulic. How to (Properly) Evaluate Cross-Lingual Word Embeddings: On Strong Baselines, Comparative Analyses, and Some Misconceptions. Proc. of the 57th ACL. 710–721.

[43] Guillaume Lample, Alexis Conneau, Marc’Aurelio Ranzato, Ludovic Denoyer, Hervé Jégou. Word translation without parallel data. 6th International Conference on Learning Representations, ICLR. 2018.

[44] Mikel Artetxe, Gorka Labaka, Eneko Agirre. Unsupervised Statistical Machine Translation. Proc. EMNLP. 3632–3642. 2018.

[45] Joulin, Armand and Bojanowski, Piotr and Mikolov, Tomas and Jégou, Hervé and Grave, Edouard. Loss in translation: Learning bilingual word mapping with a retrieval criterion. arXiv preprint arXiv:1804.07745. 2018.

[46] Ruder, Sebastian and Cotterell, Ryan and Kementchedjhieva, Yova and Søgaard, Anders. A discriminative latent-variable model for bilingual lexicon induction. arXiv preprint arXiv:1808.09334. 2018

[47] Mikel Artetxe, Sebastian Ruder, Dani Yogatama. On the Cross-lingual Transferability of Monolingual Representations. Proc. 58th ACL. 4623–4637. 2020.

[48] Elisa Bassignana, Valerio Basile, Viviana Patti. Hurtlex: A Multilingual Lexicon of Words to Hurt. Proc. 5th CLiC-it. 2018.

[49] Tomás Mikolov, Kai Chen, Greg Corrado, Jeffrey Dean. Efficient Estimation of Word Representations in Vector Space. 1st International Conference on Learning Representations, ICLR.

[50] Valerio Basile, Cristina Bosco, Viviana Patti, Manuela Sanguinetti, Elisabetta Fersini, Debora Nozza, et al. Shared Task on Multilingual Detection of Hate. SemEval, Task 5 2019

[51] Pereira-Kohatsu Juan Carlos, Quijano Sánchez Lara, Liberatore Federico, Camacho-Collados Miguel. Detecting and Monitoring Hate Speech in Twitter. Sensors. 2019. doi: 10.3390/s19214654 31717760 PMC6864473

[52] Manuela Sanguinetti and Fabio Poletto and Cristina Bosco and Viviana Patti and Marco Stranisci. An Italian Twitter Corpus of Hate Speech against Immigrants. Proc. of the 11th LREC2018. 2798–2895. 2018.

[53] Pamungkas, Endang Wahyu and Basile, Valerio and Patti, Viviana. Towards multidomain and multilingual abusive language detection: a survey. Personal and Ubiquitous Computing. 1–27. 2021.

[54] Tulika Bose, Nikolaos Aletras, Irina Illina, Dominique Fohr. Domain Classification-based Source-specific Term Penalization for Domain Adaptation in Hate-speech Detection. Proceedings of the 29th International Conference on Computational Linguistics, COLING. 6656–6666. 2022.

[55] Tulika Bose, Irina Illina, Dominique Fohr. Dynamically Refined Regularization for Improving Cross-corpora Hate Speech Detection. Findings of the Association for Computational Linguistics: ACL. 372–382. 2022.

[56] Lena Shakurova, Beata Nyari, Chao Li, Mihai Rotaru. Best Practices for Learning Domain-Specific Cross-Lingual Embeddings. Proceedings of the 4th Workshop on Representation Learning for NLP, RepL4NLP@ACL. 230–234. 2019.

[57] Alexis Conneau, Kartikay Khandelwal, Naman Goyal, Vishrav Chaudhary, Guillaume Wenzek, Francisco Guzmán, et al. Unsupervised Cross-lingual Representation Learning at Scale. Proceedings of the 58th Annual Meeting of the Association for Computational Linguistics, ACL. 8440–8451. 2020.

[58] Cañete, José and Chaperon, Gabriel and Fuentes, Rodrigo and Ho, Jou-Hui and Kang, Hojin and Pérez, Jorge. Spanish Pre-Trained BERT Model and Evaluation Data. PML4DC at ICLR 2020. 2020

[59] Dzmitry Bahdanau, Kyunghyun Cho, Yoshua Bengio. Neural Machine Translation by Jointly Learning to Align and Translate. CoRR. abs/1409.0473. 2014.

[60] Ivan Vulic, Goran Glavas, Roi Reichart, Anna Korhonen. Do We Really Need Fully Unsupervised Cross-Lingual Embeddings?. Proc. EMNLP-IJCNLP. 4406–4417. 2019.

[61] Jochen Hipp, Ulrich Güntzer, Gholamreza Nakhaeizadeh. Algorithms for Association Rule Mining—A General Survey and Comparison. SIGKDD Explor. 2000.

[62] Heikki Mannila, Hannu Toivonen, A. Inkeri Verkamo. Discovery of Frequent Episodes in Event Sequences. Data Min. Knowl. Discov. 259–289. 1997.

