# Correction: Cross-lingual hate speech detection using domain-specific word embeddings

**DOI:** 10.1371/journal.pone.0323507

**Published:** 2025-04-30

**Authors:** Ayme Arango Monnar, Jorge Perez Rojas, Barbara Poblete

The third author’s name is spelled incorrectly. The correct name is: Barbara Poblete. The correct citation is: Monnar AA, Perez Rojas J, Poblete B (2024) Cross-lingual hate speech detection using domain-specific word embeddings. PLOS ONE 19(7): e0306521. https://doi.org/10.1371/journal.pone.0306521.

There are errors in the author affiliations. The correct affiliations are as follows:

Ayme Arango Monnar^1^, Jorge Perez Rojas^2^, Barbara Poblete^1^

**1** Computer Science Department, Universidad de Chile, Santiago de Chile, Chile, **2** Cero.ai, Santiago de Chile, Chile.
